# The idiopathic preterm delivery methylation profile in umbilical cord blood DNA

**DOI:** 10.1186/s12864-015-1915-4

**Published:** 2015-09-29

**Authors:** Febilla Fernando, Remco Keijser, Peter Henneman, Anne-Marie F. van der Kevie-Kersemaekers, Marcel MAM Mannens, Joris AM van der Post, Gijs B. Afink, Carrie Ris-Stalpers

**Affiliations:** Reproductive Biology Laboratory, Academic Medical Center, University of Amsterdam, Meibergdreef 9, 1105 AZ Amsterdam, The Netherlands; Department of Clinical Genetics, Academic Medical Center, University of Amsterdam, Meibergdreef 9, 1105 AZ Amsterdam, The Netherlands; Women’s and Children’s Clinic, Department of Obstetrics and Gynaecology, Academic Medical Center, University of Amsterdam, Meibergdreef 9, 1105 AZ Amsterdam, The Netherlands

**Keywords:** Epigenetics, Methylation, UCB, Preterm, Labor, Oxytocin

## Abstract

**Background:**

Preterm delivery is the leading cause of neonatal morbidity and mortality. Two-thirds of preterm deliveries are idiopathic. The initiating molecular mechanisms behind spontaneous preterm delivery are unclear. Umbilical cord blood DNA samples are an easy source of material to study the neonatal state at birth. DNA methylation changes can be exploited as markers to identify spontaneous preterm delivery. To identify methylation differences specific to idiopathic preterm delivery, we assessed genome-wide DNA methylation changes in 24 umbilical cord blood samples (UCB) using the 450 K Illumina methylation array. After quality control, conclusions were based on 11 term and 11 idiopathic preterm born neonates. The differentially methylated positions (DMPs) specific for preterm/term delivery, neonatal sex, use of oxytocin and mode of initiation of labor were calculated by controlling the FDR p value at 0.05.

**Results:**

The analysis identifies 1855 statistically significant DMPs between preterm and term deliveries of which 508 DMPs are also attributable to clinical variables other than preterm versus term delivery.

1347 DMPs are unique to term vs preterm delivery, of which 196 DMPs do not relate to gestational age as such. Pathway analysis indicated enrichment of genes involved in calcium signalling, myometrial contraction and relaxation pathways. The 1151 DMPs that correlate with advancing gestational age (*p* < 0.05) include 161 DMPs that match with two previously reported studies on UCB methylation.

Additionally, 123 neonatal sex specific DMPs, 97 DMPs specific to the induction of labour and 42 DMPs specific to the mode of initiation of labor were also identified.

**Conclusion:**

This study identifies 196 DMPs in UCB DNA of neonates which do not relate to gestational age or any other clinical variable recorded and are specific to idiopathic preterm delivery. Furthermore, 161 DMPs from our study overlap with previously reported studies of which a subset is also reported to be differentially methylated at 18 years of age. A DMP on *MYL4*, encoding myosin light chain 4, is a robust candidate for the identification of idiopathic preterm labour as it is identified by all 3 independent studies.

**Electronic supplementary material:**

The online version of this article (doi:10.1186/s12864-015-1915-4) contains supplementary material, which is available to authorized users.

## Background

Preterm birth, defined as delivery before 37 weeks of gestation, has a global prevalence of 9.6 %. It is the leading cause of neonatal morbidity and mortality and is responsible for approximately 70 % of all neonatal deaths and 40 % of childhood neurological morbidities [1, 2]. One third of preterm births are iatrogenic because of maternal or fetal reasons, usually in relation to maternal hypertensive disease. Two thirds occur as a consequence of idiopathic preterm delivery initiated by either spontaneous preterm labor (PtLb) with intact membranes or preterm premature rupture of membranes (PPROM) [3, 4].

The initiating mechanisms behind the inappropriate early activation of labor are poorly understood. Known risk factors include uterine anomalies, multiple gestations, polyhydramnios, blood loss, infection and previous preterm birth [3]. There are no early diagnostic or prognostic markers for spontaneous preterm birth.

The timing of delivery is an intricate play between the fetus, uterus, decidua and placenta. Fetal growth increasing the tension in the uterine wall, increased oxytocin and estrogen bioactivity, progesterone withdrawal and inflammatory decidual activation, all contribute to the initiation of contractions [3, 5]. For several populations susceptibility loci influencing preterm birth have been reported, but until now this has not resulted in the identification of additional general mechanisms involved in preterm labor [6–9]. It is currently not known if there are other fetal properties, apart from fetal growth, that relate to the premature onset of delivery.

Epigenetics is an important regulatory mechanism contributing to the control of gene expression. It has been suggested that epigenetics is involved in idiopathic preterm delivery in humans [10]. DNA methylation in myometrium is known to contribute to the functional progesterone withdrawal associated with labor [11, 12]. The amnion shows significant methylation changes in the promoter region of the oxytocin receptor gene between preterm and term labor [13]. Cervical DNA methylation has also been associated with gestational length [14].

Although a few studies reported the differential DNA methylation profile of umbilical cord blood (UCB) in relation to preterm delivery, the overall emerging picture is complicated by variability in mode of delivery, limitations to specific ethnicity, the absence of information on the progression of labor after initiation of contractions and the inclusion of patients with hypertensive disease [15–17].

In the current study, we investigate the genome-wide methylation profile in UCB from 12 preterm and 12 matched term neonates all born after vaginal delivery from normotensive pregnancies and identify genes that could function as leads to establish early diagnostic markers for idiopathic preterm delivery.

## Results

### Sample cohort

We selected 24 UCB samples from neonates born after normotensive singleton pregnancies that presented headfirst and were vaginally delivered (see Additional file 1: Table S1). Gestational age at delivery ranged from 26 weeks and 3 days to 41 weeks and 4 days; delivery before 37 weeks of gestation was considered preterm. Two samples had to be excluded because of reasons described extensively below resulting in the analysis of 22 samples. Neonatal birth percentile specific for parity, neonatal sex and gestational age was calculated based on the Dutch neonatal growth charts (www.perinatreg.nl). Apart from the gestational age of delivery, neonatal weight and treatment with antenatal glucocorticoids, all characteristics intrinsic to the gestational age at birth, there are no clinical differences between groups (see Table 1).

**Table 1:** Maternal and fetal characteristics of the UCB study cohort

Clinical parameter	Preterm delivery	Term delivery	*P*-value
Number of samples	11	11	1**
Nulliparous	5 (50 %)	6 (55 %)	0.99**
Received antenatal glucocorticoids	9 (82 %)	0 (0 %)	0.0002**
Parturition initiated with spontaneous labor	7 (64 %)	7 (64 %)	1**
Parturition initiated with spontaneous rupture of membranes	4 (36 %)	4 (36 %)	1**
Labor induced or stimulated with oxytocin	5 (45 %)	7 (64 %)	0.67**
Mean gestational age at delivery in weeks + days (range)	29^+6^ (26^+3^ – 36^+4^)	39^+6^ (38^+0^ - 41^+4^)	3.83E-07*
Male neonate	5 (45 %)	6 (55 %)	0.99**
Neonatal weight (g)	1550 (990–2615)	3320 (2525–4215)	7.85E-07*
Neonatal birth weight percentile <10	0 (0 %)	3 (9.09 %)	0.21**
Maternal age	28 (19–35)	28 (19–36)	0.51*

Data are represented as numbers (%) median (range)

Abbreviations: n.s. not significant

*P*-values were calculated by unpaired student’s T-test* and Fisher’s exact test** as applicable

### Neonatal sex chromosomal variations detected by DNA methylation profiling of umbilical cord blood

**Fig. 1:**
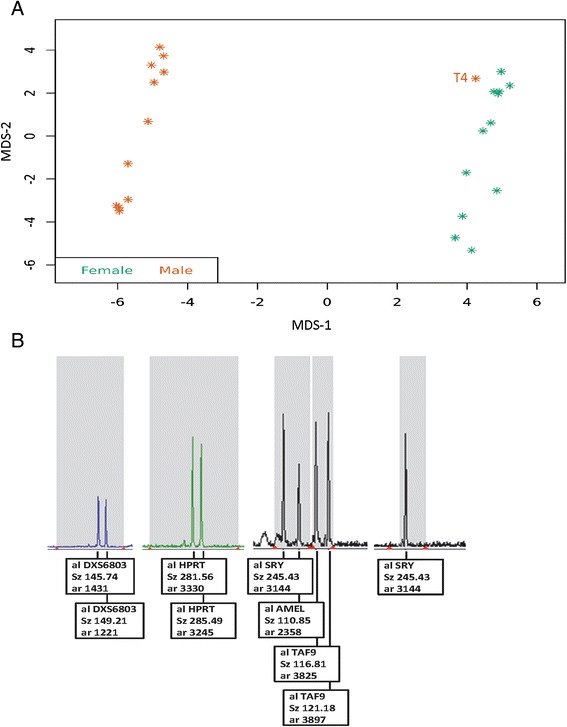
Neonatal sex chromosomal variations detected through quality control of umbilical cord blood Illumina 450 K data. **a** Multi-dimensional scaling (MDS) plot on neonatal sex on the top 1000 methylation variable positions between male (in red) and female (in green) samples; T4-Klinefilter neonate. **b** Validation of the X-chromosomal gain of neonate T4 by QF-PCR supporting the diagnosis of Klinefelter Syndrome: Markers DXS6803 (Xq21.31), HPRT (Xq26.2), TAF9L (Xq21.1), DXS1187 (Xq26.2) are specific for the X chromosome. Markers SRY (Yp11.31) and AMEL (Xp22.22)/ (Yp11.2) are specific for the Y chromosome

The Illumina 450 K BeadChip array was used to determine methylation status in our UCB cohort. As an initial check for sample integrity, the 24 samples of the original dataset were grouped as males or females based on their phenotype at birth (male: *n* = 12; female: *n* = 12; Additional file 1: Table S1) Multi-dimensional scaling (MDS) analysis was performed on raw intensity signals as a quality control step using the ChAMP package, which resulted in a male neonate born at term (T4) clustering together with the females in our dataset (Fig. 1a). Levels of probe intensity suggested that this sample gained one X chromosome. This finding was validated on DNA isolated from placenta tissue from this pregnancy that was available through our PANDA biobank project using quantitative fluorescent polymerase chain reaction (QF-PCR) (see Fig. 1b). The QF-PCR signal pattern for markers DXS6803, *HPRT*, and *TAF9* (regions Xq21.31, Xq26.2 and Xq21.1 respectively) are consistent with the presence of 2 X chromosomes. The signal from *SRY* (Yp11.31) marker confirms the presence of a Y chromosome. The signal pattern for *AMEL* (Xp22.22/Yp11.2) is consistent with two X chromosomes and one Y chromosome. The copy number analysis for chromosome X and Y showed evidence of an XXY chromosomal pattern concordant with Klinefelter syndrome. This sample was excluded from further methylation analysis (Additional file 1: Table S1).

### Genome-wide methylation differences in umbilical cord blood DNA between preterm and term infants at birth

The array quality metrics package [18] was used to check for quality of the array data and we identified one outlying sample with signal intensity strongly deviating from all other samples. This sample (PT9) was excluded from further analysis (Additional file 1: Table S1).

**Fig. 2:**
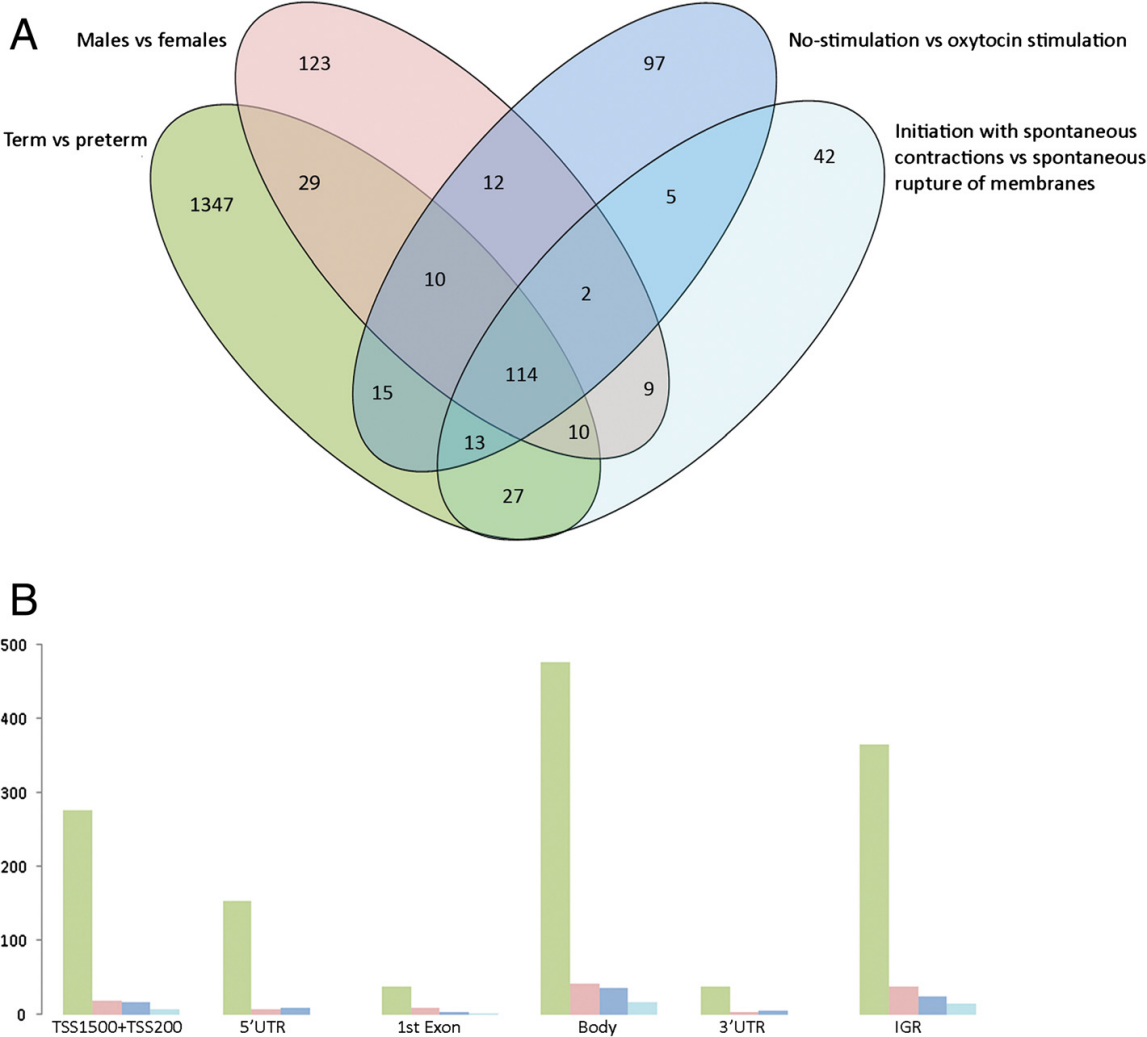
Differentially methylated positions (DMPs) in umbilical cord blood DNA between preterm and term infants. **a** Venn Diagram depicting the differentially methylated CpG sites within each of the four comparison groups (term versus preterm delivery, male versus female neonates, vaginal delivery with contraction stimulated by oxytocin versus not stimulated and whether the process of delivery initiated with PtLb versus PPROM as well as overlap between groups and subgroups). False Discovery Rate (BH: 0.05). **b** Illustration of the distribution of DMPs over gene regions for all comparison groups. The TSS200: region 200 base pairs within the transcription start site (TSS); TSS: region 1500 base pairs within the TSS excluding the TSS 200 region; UTR: untranslated region as present in the mRNA molecule respectively 5′ of the transcription start site (5′UTR) and 3′ of the termination signal (3′UTR); Body: coding and non-coding regions from the TSS until the termination codon; IGR: intergenic region

Differential methylation analysis was performed on 11 preterm and 11 term DNA samples isolated from UCB. We identified 1855 statistically significant differentially methylated positions (DMPs) (FDR *p* < 0.05), of which, 1347 DMPs can be solely attributed to methylation differences between infants born preterm and term. For the comparisons male vs female, no-stimulation vs stimulation with oxytocin and initiation with PtLb vs PPROM, the number of DMPs unique for the comparison groups are 123, 97 and 42 respectively (Fig. 2a, Additional file 2: Table S2, Additional file 4: Table S4a, Additional file 5: Table S5a, Additional file 6). The remaining 246 DMPs were shared by 2 or more groups (Fig. 2a, Additional file 7: Table S7).

For all comparison groups 35-40 % of DMPs are present in gene bodies (Fig. 2b) reflecting the distribution of probes available on the array, of which 33 % are present in gene bodies.

### Correlation of unique term vs preterm DMPs with clinical variables

**Fig. 3:**
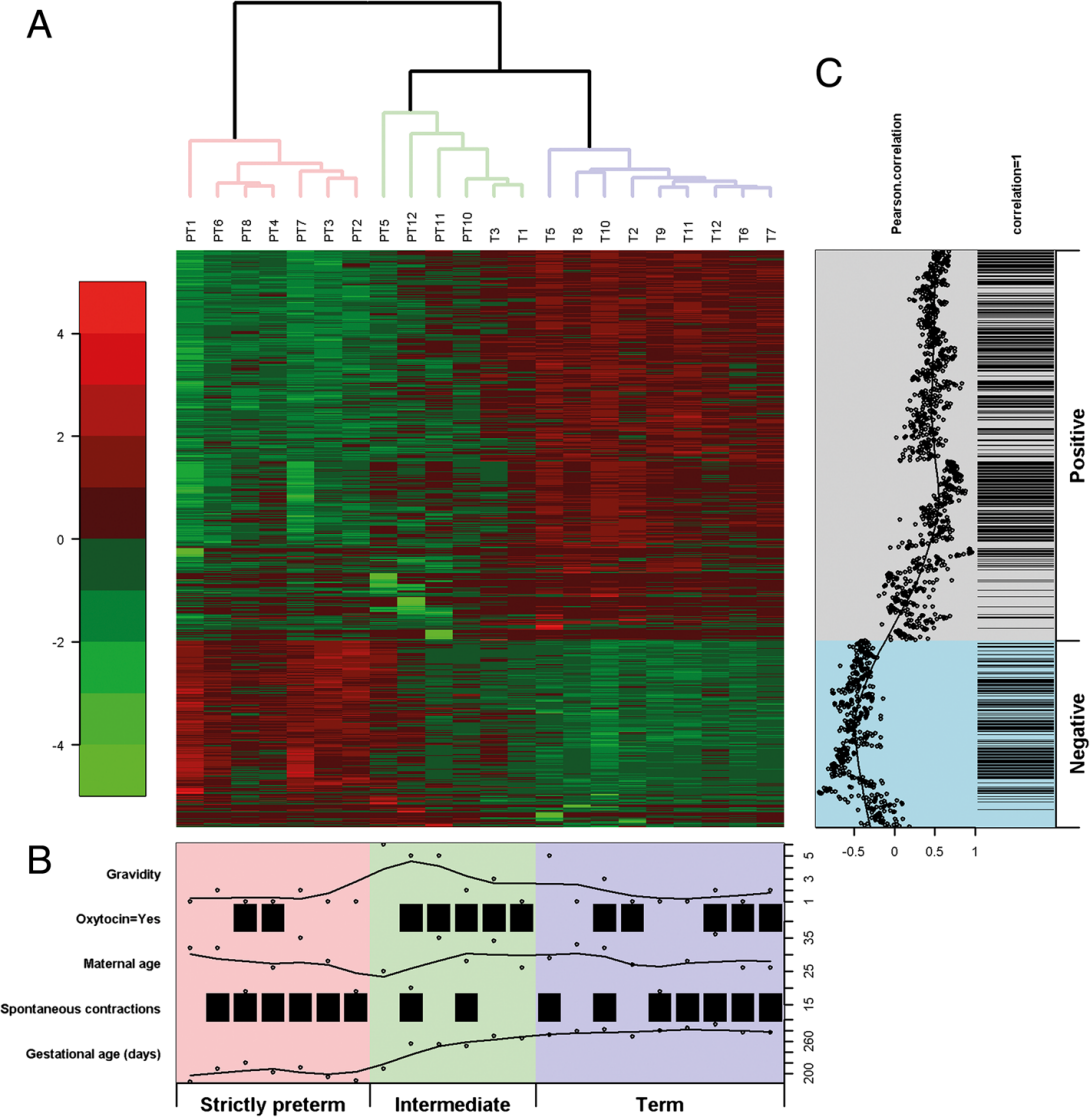
Correlation of birth DMPs with gestational age. Panel **a** Heat map illustration of the clustering of the birth DMPs with gestational age. Average linkage clustering was performed on beta values based on the correlation distance between the preterm and term group. PT1-PT12: Preterm and T1-T12: Term umbilical cord blood DNA. Panel **b** Clinical characteristics of the study cohort on the left with scaling on the right and colors reflecting the clusters on the top of panel A. Panel **c** Pearson correlation(r) with gestational age. Black bars indicate significant correlation (FDR *p* < 0.05)

A hierarchical clustering analysis was performed, in order to identify the clinical variables that associated with the 1347 DMPs identified in the term vs preterm group (Fig. 3a). The DMPs group into 3 main clusters based on gestational age; strictly preterm (26^+3^ till 31^+3^), intermediate (29^+6^ till 38^+4^) and term (38^+3^ till 41^+4^) (Fig. 3b). 1-way ANOVA with 2 degrees of freedom shows that the median gravidity of the intermediate group is significantly higher when compared to the strictly preterm group (adjusted *p* = 0.04). A Pearson correlation test was used to identify the degree of correlation of every DMP with gestational age controlling the FDR at 0.05. Of the total 1347 DMPs, 1151 DMPs correlate significantly with gestational age of which 762 DMPs with a positive and 389 DMPs with a negative correlation to gestational age (Fig. 3c). The DMPs located on gene regions with the highest correlation of gestational age with a negative correlation are *NCOR2*, *DNAJC17*, *PYCR2*, *ATP6V0A1*, *RARA* and *FBLN7* and DMPs with a positive correlation are *IGF2BP1*, *OTOF*, *ATP2B2*, *CES3* and *MYH7B*. The methylation trend of the majority of gestational age related DMPs increases with advancing gestation and within this set are 3 DMPs localising to the *ESR1* gene (see Additional file 2: Table S2, colour shading reflecting Fig. 3c).

The remaining 196 DMPs that do not correlate with gestational age reflect the systematic difference between both groups. DMPs on *KIAA0513*, *UBE21* and *AP3D1* have the highest absolute difference between groups with a p value <0.05 (see Additional file 3: Table S3a).

### Pathway analysis

Pathway analysis was performed for all the DMPs identified in each individual comparison group. The gene symbols of the observed DMPs were imported into Web-Gestalt [19, 20] and wiki pathway enrichment analysis was performed with FDR at 0.05. The 42 DMPs specific for the comparison of initiation with PtLb vs PPROM could not be assigned to specific pathways. Among the top enriched pathways in the term vs preterm UCB group, are MAPK signalling, myometrial relaxation and contraction pathway and TGF beta signalling pathway (*p* < 0.05) (Additional file 3: Table S3). The pathway involving calcium regulation in cardiac cell is observed in all 3 comparison groups. Both the term vs preterm and the male vs female comparisons showed an enrichment in myometrial relaxation and contraction pathway, insulin signalling and signalling pathways in glioblastoma. Although the pathways indicated are the same, the genes allocated to them are different, with the exception of *SLC8A1.*

### Linkage to other studies

**Fig. 4:**
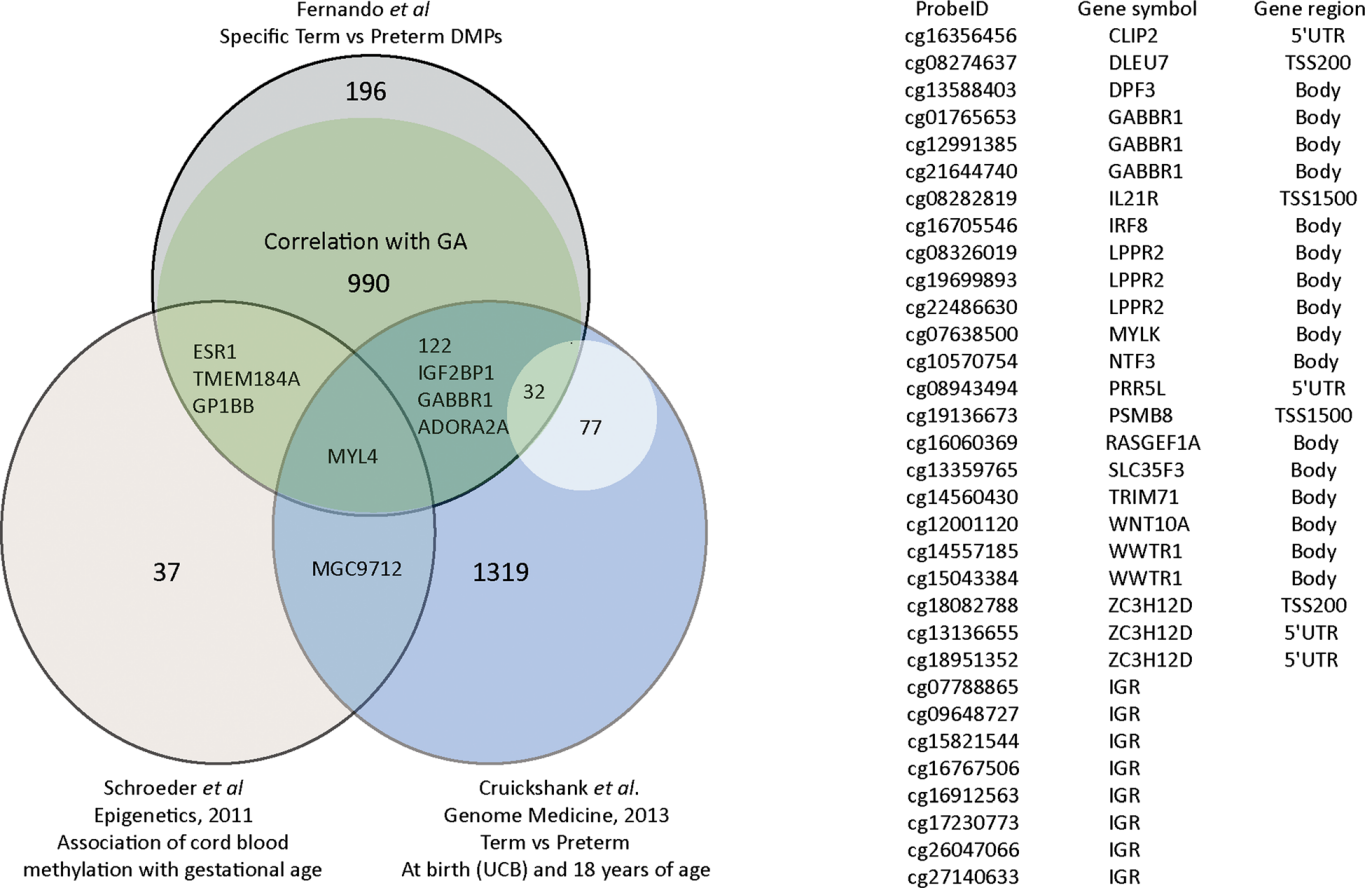
Comparison of DMPs with other study cohorts. Venn diagram with large circles reflecting the different study cohorts showing the number of individual and shared DMPs between studies. Green shading reflects all DMPs were the level of methylation correlates with gestational age in the current study. Smaller inner circle represents 109 DMPs that were observed as differentially methylated both at birth and at 18 years of age originally identified in the Cruikshank study. The current study confirms 32 of them (listed on the right)

To complement our approach of methylation analysis with relevance to preterm birth we compared DMPs identified in our study to DMPs identified by two other studies on cord blood methylation using the Illumina BeadChip technology (Fig. 4). In total, 161 of the 1347 DMPs that are uniquely assigned to the term versus preterm comparison have been reported previously [15, 17]. We identified DMPs on *ESR1*, *TMEM184A* and *GP1BB* that have been previously associated with gestational age in the Schroeder study [15]. DMPs in 157 genes, including those on *IGF2BP1*, *ADORA2A* and *GABBR1*, have been reported by Cruickshank study [17]. A single DMP on the gene encoding myosin light chain 4 (*MYL4*), in the Transcription start site 200 (TSS200) or 5′Untranslated region (5′UTR) depending on the definition of the 5′ prime end of the transcript, is identified by all 3 studies. The current study confirms 32 of the total 109 DMPs that were differentially methylated at birth and 18 years of age originally identified in the Cruikshank study. These 32 DMPs correspond to 17 genes (Fig. 4).

The 196 DMPs in the current UCB study that show no correlation with gestational age do not overlap with the 29 DMPs that associate with preterm birth adjusted for gestational age reported previously. Of these 29, the DMP cg03706951 in our study shows a negative correlation with gestational age. The other 28 are not identified as DMPs in the current study [16].

### UCB Differentially Methylated Regions (DMRs) between term and preterm neonates

**Fig. 5:**
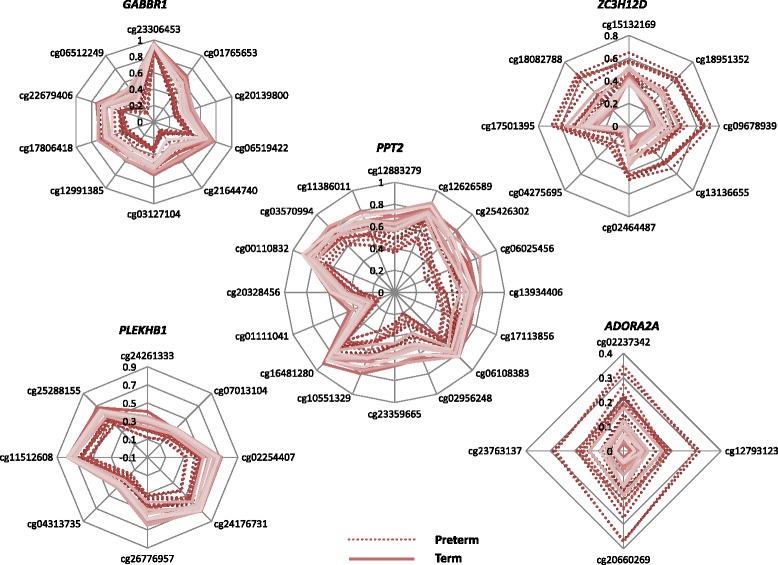
The most significant differentially methylated regions (DMRs) between cord blood of preterm and term infants at birth. Individual differentially methylated positions are listed on the outside of the circle with the radius of the circle corresponding to the level of methylation of each individual sample for each individual patient. The circle center has beta level zero. The radius of the figure corresponds to beta value with the center as beta value zero. Dotted lines represent beta values of preterm infants with every line corresponding to an individual preterm neonate. The solid lines represent beta values of term infants

With a FDR at 0.05, we identified 20 differentially methylated regions (DMRs) between preterm and term neonates; 2 non-annotated regions, a non-coding RNA (gene ID: 100131213) and 17 genes (Table 2). Out of the 20, 6 of the identified DMRs are located on chromosome 6. The genes with DMRs were queried for GO biological processes. The top 4 DMRs are located on genes involved in neuronal development such as *PPT2*, *GABBR1*, *PLEKHB1* and induction of immunity such as *ZC3H12D* [21]. Interestingly *ADORA2A*, a gene involved in vascular smooth muscle contraction pathway, was identified as a DMR covering 4 DMPs in this analysis (Fig. 5).

**Table 2:** Gene annotations of differentially methylated regions in UCB DNA from term and preterm born neonates

Gene ID	Gene symbol	Number of DMPs	Adjusted p value	Chromosomal location	DMR start position	DMR end position
9374	PPT2	16	1,78E-12	6p	32120761	32121161
780	DDR1	13	9,46E-06	6p	30853990	30854352
2550	GABBR1	10	4,15E-08	6p	29599012	29599538
58473	PLEKHB1	8	7,08E-09	11q	73356997	73357675
340152	ZC3H12D	8	1,13E-06	6q	149805798	149806410
57224	NHSL1	7	2,21E-07	6q	138866338	138867573
23007	PLCH1	6	3,45E-07	3q	155421691	155422249
3200	HOXA3	5	7,80E-05	7p	27153536	27153782
79899	PRR5L	5	2,08E-07	11p	36422098	36422894
135	ADORA2A	4	1,68E-05	22q	24823473	24823635
3141	HLCS	4	1,46E-05	21q	38362713	38362783
2247	FGF2	3	1,52E-06	4q	123747468	123747623
51199	NIN	3	1,25E-06	14q	51296493	51296731
5696	PSMB8	3	6,23E-07	6p	32812868	32813030
56963	RGMA	3	9,13E-07	15q	93617020	93617110
10418	SPON1	3	1,83E-07	11p	13983766	13983811
202915	TMEM184A	3	1,95E-06	7p	1595968	1596132

## Discussion

DNA methylation plays an important role in gene regulation and in the etiology of complex diseases [22]. UCB DNA is an easily available source and relevant for the fetal state at the moment of birth. The umbilical cord blood DNA comprises of a mixed population of all types of blood cells and other tissue cells that have entered the circulation. However, the extent to which the DNA methylation profile of UCB reflects those in other tissues is still a matter of debate. Fetal DNA is detectable in the maternal circulation early in pregnancy facilitating non-invasive prenatal testing [23, 24]. Recent developments have shown that the fetal methylome is also accessible from maternal plasma, thus enhancing the opportunity to determine fetal epigenetic markers in the maternal circulation [25].

In the current study, we characterize the methylation profile in UCB DNA specific to spontaneous preterm delivery, neonatal sex, use of oxytocin and mode of initiation of labor using the Illumina 450 K bead chip. This technical approach is also evidently able to reveal copy number aberrations, as we encountered a 47, XXY neonate in our sample cohort.

There is evidence suggesting gender-specific differences to the susceptibility and progression of some diseases [26–28]. Some studies have shown that preterm born females have a better survival rate and neurological outcome compared to preterm males [29–32]. There is no functional data proving epigenetics as causal to gender differences but the current study indicated some putative associations. We identified 123 autosomal DMPs that differ between males and females irrespective of the preterm/term status. Within this group are DMPs located on *PRKCZ*, *GBE1 and GYG. PRKCZ* in cord blood T cells has been associated with allergy risk in infants and it has been reported that gender differences affect susceptibility to the development of hypersensitivity reactions [33, 34]. The *PRKCZ* gene has also been linked to development of bipolar disorder [35] in line with the increased risk of psychiatric illness such as bipolar disorder in males compared to females [36, 37].

Similarly we also established DMPs in certain CpG sites specific to oxytocin usage to induce or stimulate labor. Some of the DMPs in the oxytocin vs no-oxytocin group are *CACNA1D*, *ADCY9 and ITGA3.* According to KEGG pathways both *CACNA1D* and *ADCY9* are linked to the oxytocin signalling pathway. In-vivo studies show that α3β1 integrin (*ITGA3*) mediated signalling contributes to the control of amplitude and duration of contractions in response to oxytocin in mammary myoepithelial cells [38].

The number of DMPs specific to spontaneous contractions vs spontaneous rupture of membranes comparison is too small to perform pathway analysis.

The majority of methylation differences in our study cohort are between preterm versus term pregnancies. The 1347 DMPs solely attributable to term versus preterm birth were mainly observed in gene bodies, intergenic regions and within 1500 basepairs of the transcription start site, reflecting the number of probes present in these regions. About 85 % of the 1347 DMPs observed in UCB of neonates born term vs preterm have a clear relation with advancing gestational age. This has been reported before and is assumed to reflect the haematological changes as leucocyte content, nucleated reticulocyte content and maturation of red blood cells, all of which strongly correlate with gestational age [39–42]. In the current cohort gestational ages range from 26^+3^ to 41^+4^ weeks. Hierarchical clustering of the 1347 DMPs separates early preterm from term deliveries and identifies an intermediate gestational age group (29^+6^ – 38^+4^) with a statistically significant higher gravidity. The remaining 196 DMPs represent which do not correlate with gestational age represent systematic differences between the preterm and term born neonates and have not been reported previously in relation to preterm delivery. A study interrogating methylation patterns in UCB DNA from African-Americans identified 29 CpG sites associated with preterm birth adjusted for gestational age. None of those were corroborated by the current study [16]. Wiki pathway analysis and gene enrichment of our set of 196 DMPs shows calcium and TGF beta signalling, myometrial contraction and relaxation pathways and the corticotrophin releasing hormone pathway. It is well established that calcium signalling is crucial in the human myometrium for initiation of labor [43]. The genes which are represented in the myometrial contraction and relaxation pathways are sodium and calcium exchanger *SLC8A1,* which also plays a role in cardiomyocyte contractions, chemokine receptor *CXCR7*, guanine nucleotide binding protein *GNG7* and transmembrane signalling enzyme phospholipase C *PLCG2* respectively. Further research will have to determine if they are putative foetal biomarkers for idiopathic preterm delivery that can be analysed from the maternal circulation.

Ideally high-thoughput data from clinical samples need to be validated either by additional experiments or comparative validation to other studies. The limitation of the current study is that we have not experimentally validated this finding. However, we have approached this issue with detailed comparison of previously published independent studies on UCB methylation. There have been 3 previous reports on DNA methylation profiles in UCB analysed with respect to gestational age at delivery using the Illumina platform [15–17]. The study by Schroeder et al*.* analysed 453 UCB samples with gestational age ranging from 32 to 43 weeks of gestation on a 27 K platform. Results were adjusted for a number of clinical variables including neonatal sex and parity and they showed that associations were independent of the method of delivery or induction of labor. The study by Cruickshank et al*.*, interrogated both UCB and blood at 18 years of age of 12 preterm (25 to 30 weeks of gestation) and 12 term (36–42 weeks of gestation) born neonates using the 450 K platform. Two thirds of the mothers did not experience labor. For both these studies gravidity was not reported.

The 161 DMPs which overlap between our study and the above mentioned studies provide increased evidence of presence of these methylation differences in UCB of preterm and term infants at birth. The overlapping DMPs on *ESR1* and *MYL4* are of interest in the context of preterm delivery. Increased *ESR1* (estrogen receptor) gene expression in the myometrium is triggered by functional progesterone withdrawal and results in increased estrogen bioactivity, an important contributor to the transition from myometrial quiescence to synchronised contractions [44, 45]. As all our neonates were delivered vaginally, we cannot exclude the fact that labor is associated with the differential methylation of the *ESR1* gene. However, based on the study of Schroeder which also included samples from neonates delivered by caesarean section, this seems unlikely.

*MYL4* encodes myosin light chain 4 that is essential to the myometrial contraction pathway. *MYL4* expression in mice myometrium is downregulated over two-fold during quiescence indicating an active role in the progression to myometrial contractions [46].

A single DMP (cg19817652) on *C17orf98* identified by the Cruickshank study as relating to gestational age is uniquely attributable to neonatal sex in our study. The 32 overlapping DMPs with Cruickshank study which has been experimentally validated to be present at 18 years of age is of significance due to the long-term consequence of preterm birth.

DMR analysis identifies 20 significant differentially methylated regions. The most significant DMR is located on the *PPT2* gene on chromosome 6p21.3 and covers 16 DMPs. *PPT2* is located on the major histocompatibility locus (MHC-III) and its deficiency in homozygous knock out mice causes a neurodegenerative disorder [47]. There are 2 other DMRs present on 6p21.3 corresponding to *DDR1* with 13DMPs and *PSMB8* with 3DMPs. *ZC3H12D* activates TLR signalling in macrophages and plays a role in immunity and inflammatory diseases. The inflammatory pathway is a well-established important contributor to the initiation of synchronised myometrial contractions and interestingly the differential methylation of *ZC3H12D* has been shown to persist until at least the age of 18 years [48]. The *ADORA2A* encodes the adenosine A2a receptor, a member of the G-protein coupled receptor superfamily. Interleukin-1b activates myometrial inflammation and is able to up regulate *ADORA2A* expression >20 fold in primary culture of pregnant human uterine myocytes [49]. *ADORA2A,* also reported by the Cruickshank study, shows a relative increased methylation status in UCB DNA of neonates born after idiopathic preterm labor.

Comparing the methylation and gene expression profiles of myometrium, placenta and umbilical cord blood of preterm and term deliveries could shed more light on the functional relevance of the methylation changes observed in the cord blood DNA.

## Conclusion

This study identifies 1347 methylation changes in umbilical cord blood specific to idiopathic preterm delivery. The approach to methylation analysis in this study also delineates methylation changes specific to neonatal sex giving more insight into sex-specific autosomal methylation differences at birth enhancing the understanding of gender specific susceptibility to a specific disease.

In addition, the identification of 161 DMPs which overlap with other studies offer supportive evidence of robust methylation differences in UCB between preterm and term infants. Meta-analysis of raw data sets of these independent studies combined with detailed information on clinical data will provide a more robust set of DMPs which can be used as diagnostic markers for spontaneous preterm delivery.

## Methods

### Study design

From the Preeclampsia And Non-preeclampsia Database (PANDA) project we used placenta tissue and umbilical cord genomic DNA with clinical data on maternal health, the course of pregnancy and pregnancy outcome with informed consent, approved by the ethics committee of the Academic Medical Center Hospital of the University of the Amsterdam.

We selected 24 umbilical cord blood (UCB) samples from vaginal deliveries of normotensive singleton pregnancies where the live born neonate presented head first and the membranes ruptured spontaneously, either before or after the start of contractions. Pregnancies with maternal diabetes, hyper- or hypothyroidism, preeclampsia, HELLP syndrome, congenital heart disease, or intrahepatic cholestasis of pregnancy, intra-uterine growth retardation, fetal distress or any kind of infection were excluded. We selected UCB from 12 neonates delivered <37 weeks of gestation and they were matched to 12 neonates delivered ≥ 37 weeks of gestation with respect to parity, maternal age, use of oxytocin, initiation of parturition with spontaneous labor or rupture of membranes and neonates small for gestational age. UCB was collected in EDTA tubes and processed by Gentra Autopure LS98™ system (Gentra Systems). Table 1 illustrates the maternal and fetal characteristics of the cord blood study cohort (Additional clinical data in Additional file 1: Table S1).

### Sample preparation and methylation assay

For each sample, 1ug of genomic DNA was bisulphite converted using the EZ-DNA methylation kit (Zymo Research, D5001). The bisulphite converted DNA was subjected to whole genome amplification, fragmentation followed by hybridization on Infinium Illumina 450 K BeadChip array and Cy3 or Cy5 signal intensities were generated using Illumina iScan.

### Quantitative fluorescence-PCR analysis

DNA was isolated from a placenta biopsy using the MagnaPureLC DNA isolation kit II (Roche). For QF-PCR aneuploidy screening the QST*Rplus V2 kit (Elucigene Diagnostics, AN0PLB2) was used according to the manufacturer’s instructions. The amplified sample was analyzed with the ABI 3500 (Applied Biosystems, Foster City, California, USA). Interpretation of results was performed using guidelines from the manual, the 2012 ACC/CMGS ‘QF-PCR for the diagnosis of aneuploidy best practice guidelines’ V3.01, 22 and the CCMG ‘Practice Guidelines for Prenatal QF-PCR’.

### Initial data analysis

The array quality metrics package was used to check for outliers based on array quality that were subsequently excluded from further analysis [18]. Raw data files containing the methylation intensities were imported into the ChAMP package [50]. Additional quality control was performed using ChAMP, and probes with a detection p-value less than *p* < 0.01 were filtered out and used for the analysis scores. The X and Y chromosome methylation levels in UCB were checked against gender of the neonate before they were removed from the analysis altogether. One sample from the preterm group (PT9) was identified as an outlier based on the distance between arrays and intensity distributions. This sample was excluded from the dataset before normalization. The Beta-mixture quantile normalization (BMIQ) method was used to normalize the dataset and to correct for the Illumina assay-II signal bias within the Illumina 450 K BeadChip [50]. To avoid methylation bias due to cross hybridizing probes, 29,233 probes that were previously reported to cross hybridize were removed from the dataset [51]. After the quality control steps, differential methylation analysis was performed with the remaining 443,190 probes.

### Statistical testing

Linear regression modelling algorithms from the LIMMA package [52] were used to construct linear models for each comparison group which include neonatal sex, type of parturition initiation and induction or co-stimulation with oxytocin. Samples were assigned into 4 comparison groups based on 1) preterm or term status 2) neonatal sex: male or female 3) whether oxytocin was administered at any time before delivery 4) whether parturition initiated with PtLb or PPROM. Models were constructed by setting the M value (log transformed beta value) as outcome and the attributed group phenotype as independent variable for each group. The decideTest function was used for multiple testing across all groups. The differentially methylated positions (DMPs) were called based on the p value generated by multiple testing after a false discovery rate correction (FDR) according to Benjamini and Hochberg *p* < 0.05. Gene region information was obtained from the Illumina manifest file version 1.2. After identifying the 1347 DMPs that were exclusive to the preterm vs. term comparison group they were further used to define differentially methylated regions (DMRs) and perform pathway analysis.

### Differentially methylated regions

DMRs were calculated using the probe lasso function of the ChAMP package [50]. A gene region was called DMR if it had a minimum of 3 or more significant probes after adjusting for FDR (*p* < 0.05) and a neighbouring DMR is separated by a distance of maximum 1000 base pairs.

### Pathway analysis

The DMPs for each comparison group were narrowed down to their gene names and imported into Web-Gestalt for wiki-pathway analysis [19, 20] A hypergeometric statistical testing was performed and a p value was generated after FDR correction (Benjamini and Hochberg) *p* = 0.05.

### Availability of supporting data

The methylation data discussed in this publication have been deposited in NCBI’s Gene Expression Omnibus [53] and are accessible through GEO Series accession number GSE66459 (http://www.ncbi.nlm.nih.gov/geo/query/acc.cgi?acc=GSE66459).

## Additional files

Additional file 1:
**Patient data.** (XLS 104 kb) Additional file 2:
**DMPs correlating to gestational age (refers to Fig.** **3c** **).** (XLSX 140 kb) Additional file 3:
**a. Systematic differences (DMPs) Term vs Preterm. b. Term vs Preterm-wikipathways.** (ZIP 99 kb) Additional file 4:
**a. Systematic differences (DMPs) Female vs Male neonates. b. Females vs Males- wikipathways.** (ZIP 30 kb) Additional file 5:
**a. Systematic differences (DMPs) Oxcytocin use. b. Oxcytocin use-wikipathways.** (ZIP 25 kb) Additional file 6:
**Systematic differences (DMPs) Initiation with spontaneous PtLb vs PPROM.** (XLS 44 kb) Additional file 7:
**DMPs shared between groups.** (XLS 155 kb)

## Abbreviations

DMP: Differential methylated position
DMR: Differentially methylated region
FDR: False discovery rate
GO: Gene ontology
IGR: Intragenic region
MDS: Multiple dimensional scaling
QF-PCR: Quantitative fluorescent polymerase chain reaction
TSS1500: Within 1500 basepairs of the transcription start site
TSS200: Within 200 basepairs of the transcription start site
UCB: Umbilical cord blood
UTR: Untranslated region
